# Age-dependent effects of metformin on human oligodendrocyte lineage cell ensheathment capacity

**DOI:** 10.1093/braincomms/fcae109

**Published:** 2024-03-28

**Authors:** Abdulshakour Mohammadnia, Qiao-Ling Cui, Chao Weng, Moein Yaqubi, Milton G F Fernandes, Jeffery A Hall, Roy Dudley, Myriam Srour, Timothy E Kennedy, Jo Anne Stratton, Jack P Antel

**Affiliations:** Neuroimmunology Unit, Montreal Neurological Institute and Department of Neurology and Neurosurgery, McGill University, Montreal H3A 2B4, Canada; Neuroimmunology Unit, Montreal Neurological Institute and Department of Neurology and Neurosurgery, McGill University, Montreal H3A 2B4, Canada; Neuroimmunology Unit, Montreal Neurological Institute and Department of Neurology and Neurosurgery, McGill University, Montreal H3A 2B4, Canada; Department of Neurology, Renmin Hospital of Wuhan University, Wuhan, Hubei 430060, P.R. China; Neuroimmunology Unit, Montreal Neurological Institute and Department of Neurology and Neurosurgery, McGill University, Montreal H3A 2B4, Canada; Neuroimmunology Unit, Montreal Neurological Institute and Department of Neurology and Neurosurgery, McGill University, Montreal H3A 2B4, Canada; Department of Neurosurgery, McGill University Health Centre and Department of Neurology and Neurosurgery, Montreal H3A 2B4, Canada; Department of Pediatric Neurosurgery, Montreal Children’s Hospital, Montreal H4A 3J1, Canada; Division of Pediatric Neurology, Montreal Children’s Hospital, Montreal H3A 2B4, Canada; Neuroimmunology Unit, Montreal Neurological Institute and Department of Neurology and Neurosurgery, McGill University, Montreal H3A 2B4, Canada; Neuroimmunology Unit, Montreal Neurological Institute and Department of Neurology and Neurosurgery, McGill University, Montreal H3A 2B4, Canada; Neuroimmunology Unit, Montreal Neurological Institute and Department of Neurology and Neurosurgery, McGill University, Montreal H3A 2B4, Canada

**Keywords:** age groups, metformin, nanofibre ensheathment, oligodendrocyte lineage

## Abstract

Metformin restores the myelination potential of aged rat A2B5+ oligodendrocyte progenitor cells and may enhance recovery in children with post-radiation brain injury. Human late progenitor cells (O4+A2B5+) have a superior capacity to ensheath nanofibres compared to mature oligodendrocytes, with cells from paediatric sources exceeding adults. In this study, we assessed the effects of metformin on ensheathment capacity of human adult and paediatric progenitors and mature oligodendrocytes and related differences to transcriptional changes. A2B5+ progenitors and mature cells, derived from surgical tissues by immune-magnetic separation, were assessed for ensheathment capacity in nanofibre plates over 2 weeks. Metformin (10 µM every other day) was added to selected cultures. RNA was extracted from treated and control cultures after 2 days. For all ages, ensheathment by progenitors exceeded mature oligodendrocytes. Metformin enhanced ensheathment by adult donor cells but reduced ensheathment by paediatric cells. Metformin marginally increased cell death in paediatric progenitors. Metformin-induced changes in gene expression are distinct for each cell type. Adult progenitors showed up-regulation of pathways involved in the process of outgrowth and promoting lipid biosynthesis. Paediatric progenitors showed a relatively greater proportion of down- versus up-regulated pathways, these involved cell morphology, development and synaptic transmission. Metformin-induced AMP-activated protein kinase activation in all cell types; AMP-activated protein kinase inhibitor BML-275 reduced functional metformin effects only with adult cells. Our results indicate age and differentiation stage-related differences in human oligodendroglia lineage cells in response to metformin. Clinical trials for demyelinating conditions will indicate how these differences translate *in vivo*.

## Teaser

Mohammadnia *et al.* demonstrate distinct age-related functional and molecular responses of human brain-derived mature and A2B5+ oligodendrocyte lineage cells to metformin. Metformin *in vitro* enhances nanofibre ensheathment by adult-derived cells while inhibiting responses of paediatric samples. Clinical trials in demyelinating conditions will indicate how these differences translate *in vivo*.

## Graphical Abstract

**Graphical abstract:**
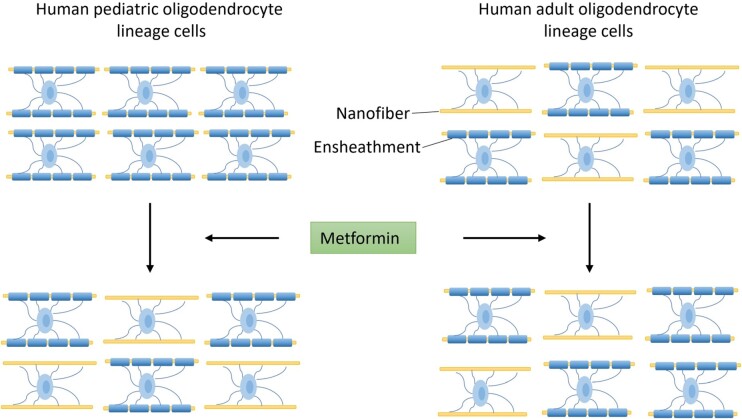

## Introduction

Restoration of myelin remains a major objective for recovery of neurologic function in an array of acquired insults of the central nervous system across the age spectrum. Multiple sclerosis is the prototype disorder in adults. The extent of remyelination is most apparent in acute lesions (relapsing phase) and very limited in more chronic lesions (progressive disease phase) and with ageing.^1-3^ White matter injury in the paediatric age group includes hypoxic-ischaemic encephalopathy in the very young and the effects of radiation.^4,5^  *In vivo* and *in vitro* studies in animals indicate that myelination capacity decreases with age, reflecting intrinsic properties of the oligodendroglia (OL) lineage cells (differentiation and migration)^6^ and the influence of positive and negative signals received from the local environment.^7^

The precise OL lineage cells mediating remyelination remains to be defined. Most experimental data derived from models in which mature myelinating OLs are depleted, demonstrate that recruitment of progenitor cells is essential; other models indicate the potential for surviving previously myelinating OLs to participate.^8^ A population of OL lineage cells, recognized by the anti-ganglioside antibody A2B5+ cells can be derived from the human central nervous system and are shown to restore myelin when transplanted into shiverer mice.^9^ These ‘progenitor’ cells co-express O4 indicating they are relatively late in the OL development lineage and have been referred to as ‘pre-OLs’.^10-12^ We previously observed that these cells derived from surgical resections were superior to mature OLs with regard to ensheathment of nanofibres.^13^ Cells from paediatric sources were superior to those from adults (age range 30–60 years) in terms of their ensheathment capacity, an effect that could be attributed to age-related intrinsic changes and their state of maturation, as determined by RNA sequencing.^13^

Metformin is reported to have a positive effect on both the injury and repair phases of adult experimental demyelinating models.^14-18^ Neumann *et al.*, using A2B5+ progenitor cells derived from 2 to 3 month old versus aged rats, showed the reduced age-related capacity of such cells to repair ethidium-bromide-induced central nervous system demyelinated lesions.^19^ The age-linked deficit could be reversed with metformin, was dependent on signalling via AMP-activated protein kinase (AMPK) and reproduced what had been observed with starvation conditions. Metformin is now under study for its potential to promote myelin repair in progressive multiple sclerosis (www.mssociety.org.uk). Ayoub *et al.*^4^ reported a potential positive effect of metformin on recovery from radiation-induced central nervous system injury in children in a pilot clinical trial, attributing the effect to stimulation of endogenous neural precursor cells within the hippocampus. Conversely, metformin applied to glioma cells reduces cell growth, with effects attributed both to direct impact on tumour cells (reduced proliferation, apoptosis) and modulation of properties of surrounding microglia.^20,21^

The aim of the current study was to assess the effects of metformin on human OL lineage cell (A2B5+ progenitors and mature OLs) capacity to ensheath nanofibres. We present the results of our functional assays indicating the discrepant age-related results and a molecular analysis identifying the potential basis for the results.

## Materials and methods

### Study population

Functional ensheathment studies were conducted on cells isolated from 10 adult samples (ages 23–68 years) and 14 paediatric hospital-derived samples (ages 1.5–18 years). The latter samples were sub-grouped into ages under 12 years [referred to as paediatric young (PedY) and 13–18 years (referred to ‘adolescent’ (Adl)]. Additional cases were used for studies as indicated in Supplementary Table 1. All patients presented with epilepsy. Histological diagnosis for most paediatric cases was focal cortical dysplasia; for adult cases—focal cortical dysplasia and gliosis (one metastatic tumour case). As described in detail by Luo *et al.*,^13^ tissue samples used for the studies were collected from ultrasonic surgical aspirator bags containing ‘surgical corridor’ material that was as separate from sites of pathology as possible. Adult surgical samples were obtained from the Department of Neuropathology at the Montreal Neurological Institute and Hospital. Samples of cases up to 18 years were obtained from the Montreal Children’s Hospital. The use of adult tissues was approved by the Montreal Neurological Institute Neurosciences Research Ethics Board and the use of paediatric tissues by the Montreal Children’s Hospital Research Ethics Board.

### Cell isolation

Immediately *ex-vivo* (1–2 h) samples were subjected to trypsin digestion followed by Percoll gradient centrifugation as previously described.^13^ To obtain enriched populations of mature OLs and A2B5+ cells, the total dissociated cell sample was cultured overnight in Dulbecco's Modified Eagle Medium (DMEM)/F12 medium (Sigma, Oakville, ON, USA) containing N1 supplementary media (Sigma), 0.01% bovine serum albumin (Sigma); the floating cells were then collected, leaving behind the adherent microglia. A2B5 antibody conjugated microbeads (Miltenyi Biotec, Auburn, CA, USA) were then used to select A2B5+ cells, also referred to as A+; these comprise ∼5–10% of the total OL lineage fraction. The non-selected cells comprise the mature OL fraction and are referred to as A-cells. As previously documented, these cell preparations are devoid of astrocytes and neurons and have <5% microglia.^11,13^ The mean % O4+ cells in dissociated cell cultures were the following: adult cells A+ 93.5 ± 1.0%; A− 91.5 ± 1.4%, *n* = 13; paediatric cells A+ 92.8 ± 1.0%, A− 89.9 ± 0.9%, *n* = 13.

### Cell culture studies—nanofibre ensheathment

Aliquots of A2B5+ and A2B5− cells were plated in multi-well aligned nanofibre plates (The Electrospinning Company Ltd., Didcot, Oxfordshire, UK) (1 × 10^5^ cells per microwell) and cultured in DMEM/F12+ N1 medium supplemented with B27 (Life Technologies, Grand Island, NY, USA) and T3 for 2 weeks. Cell cultures were immunolabelled with O4 antibodies (R&D Systems, Oakville, ON, USA) followed by corresponding secondary antibodies conjugated with either Alexa Fluor 647 or 488 (Thermo Fisher Scientific) or Texas Red (Biosource, Camarillo, CA, USA). To determine the percentage of O4+ cells in a culture ensheathing a nanofibre, a blinded rater counted individual O4+ cells whose processes could be identified as being connected to their cell bodies (4',6-diamidino-2-phenylindole staining of nuclei). Ensheathed processes were recognized by their straight alignment with underlying nanofibres and increased thickness of the segments (illustrated in Supplementary Fig. 1A–C). Cells were assigned as ensheathing (one or more segments) or not and counted by Plugin Cell Counter ImageJ program.

### Dissociated cell cultures

A2B5+ and A2B5− cells were plated into 96-well poly-L-lysine and extracellular matrix-coated chamber slides (30 000 cells per well) in the same defined medium as for the ensheathment assays. Experimental studies were conducted over the subsequent 6 days. Cell properties assessed included expression of O4 and cell viability (live staining with propidium iodide).

**Figure 1:**
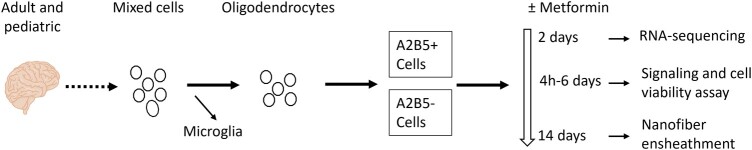
Schematic overview of our cell isolation and experimental procedures employed to investigate the impact of metformin on the human OL lineage cells.

Metformin (10 µM; Sigma) was added to selected nanofibre or dissociated cell cultures every other day. H_2_O was used as vehicle control for metformin experiments. Figure 1 presents a schematic outline of our cell isolation and experimental procedures.

### Statistical analyses

For all cell culture studies, we performed each experiment with at least three independent replications (specified in figure legends). In all the imaging analyses, an observer who was blinded to the experimental groups conducted the quantification. The data were analysed using GraphPad Prism 9.1 software and are presented as means ± SEM. All data were derived from means of three or more individual patient samples, i.e. the number of data points for each group analysed in these plots are the number of individual sample donors. Student’s *t*-test was used for comparisons between the two groups. Comparison between three or more independent groups were performed using one-way analysis of variance, followed by Sidak’s *post hoc* test. *P*-values <0.05 were considered statistically significant.

### RNA sequencing and bioinformatic analysis

RNA sequencing—RNA was extracted from dissociated cultures of A2B5+ or A2B5− cells after 2 days in culture under control conditions or with the addition of metformin. Bulk sequencing studies were conducted on three or four samples from each of the age groups, as indicated in Supplementary Table 1. RNA sequencing was done at the Génome Québec Centre with the Illumina platform and NovaSeq 6000 PE100 machine. The GenPipes workflow was used for alignment to the GRCh38 human genome and read counting. Briefly, raw FastQ files were aligned to the GRCh38 genome reference using STAR aligner with default parameter^22^ and raw reads were quantified using HTseq count. We employed several steps in our analysis to address the issue of heterogeneity in gene expression profiles among different human patient samples. Raw read counts were normalized, variance-stabilized and transformed using the DESeq2 package in R.^23^ Instead of comparing differentially expressed genes between populations of samples using DESeq2, we took advantage of having paired samples (treated versus untreated) for each patient. We performed paired *t*-test analysis to account for the effect of metformin treatment within each pair of samples. This approach allowed us to address the substantial heterogeneity between different samples, which vary in terms of age and sex. By using paired *t*-test analysis, we calculated *P*-values for each of the four groups treated with metformin (N1 versus metformin). Genes that exhibited consistent increasing or decreasing patterns across samples, with *P*-values <0.05, were considered as differentially expressed genes.

Pearson correlation was used to calculate column and row distances and pairwise average linkage was used for clustering. Heatmaps were generated after clustering using the Hierarchical Clustering Image function in GenePattern (v3.9.11).^24^ Gene ontology analysis was done with the significantly differentially expressed genes (fold change > 1.2, *P* < 0.05) with minimum normalized read counts >50 expressed genes using Enrichr. Pathways with a *P*-value <0.05 were considered enriched.^25,26^ Single sample gene set enrichment analysis implanted in GenePattern^24^ was used to provide pathway-level enrichment in bulk RNA-seq data. C5.go.bp.v2022.1.Hs.symbold.gmt which contains 7763 gene sets derived from the gene ontology biological process ontology was used to conduct single sample gene set enrichment analysis. Top pathways involved in lipid metabolism and mitochondria were filtered based on the *P*-values to generate heatmaps. The top 100 marker genes for oligodendrocyte progenitor cells obtained from our local single-cell RNA-seq datasets (Yaqubi^26^) were used to identify OL lineage genes affected upon metformin treatment in paediatric A2B5 positive cells. All bulk RNA-seq samples utilized in this study have been deposited in the Gene Expression Omnibus of National Center for Biotechnology Information (NCBI) with GSE247159 number.

In addition to our local datasets, data from Neuman *et al.*^19^ (GSE134765 accession number) was downloaded from Gene Expression Omnibus. This contains data on three adult rats (age 20–24 months) A2B5+ cell samples. Dataset was normalized, variance-stabilized, transformed and clustered with the same protocol as mentioned for our datasets.

### Measurement of AMPK activation

Immunofluorescence—for these studies, metformin-treated (2–6 days) and control cells in dissociated cultures were immunostained with anti-phosphorylated AMPK (pAMPK) antibody (5 µg/ml, Invitrogen #701068) and O4 antibodies. Images were acquired with a Zeiss fluorescence microscope. The intensity of immunofluorescence of pAMPK in each O4+ cell was measured with ImageJ and expressed as a mean grey value.

Western blot—Protein concentrations were determined using bicinchoninic acid protein assay dye reagents (Pierce, Thermofisher, #23225) as described by the company. Sodium dodecyl-sulfate polyacrylamide gel electrophoresis was run with 20 µg of protein in each sample in 10–15% acrylamide gels. Proteins were transferred to a polyvinylidene difluoride membrane. Membranes were probed with the indicated antibodies [pAMPK (1:1000), total AMPK (1:1000) and actin (1:1000, Cell Signalling #9957)] overnight at 4°C. Visualization was done with horseradish peroxidase-conjugated secondary antibody used in conjunction with an ECL Western blot detection kit (Cell Signalling #12630S). The blots were quantified with the Gels program in ImageJ, and the value of the pAMPK was corrected with total AMPK or total actin.

As ‘positive’ controls for induction of pAMPK, a nutrient and energy sensor that maintains energy homeostasis,^27^ we utilized hOLs that had been maintained under metabolic stress conditions comprised of low glucose (LG) or no glucose (NG)/reduced nutrient conditions as previously described.^28,29^ In additional studies, the AMPK inhibitor BML-275/Dorsomorphin (Santa Cruz Biotechnology, CAS 866405-64-3) was added to metformin-treated and control nanofibre cultures.

## Results

### Functional studies on nanofibre ensheathment by A2B5+ and A2B5− cells

**Figure 2:**
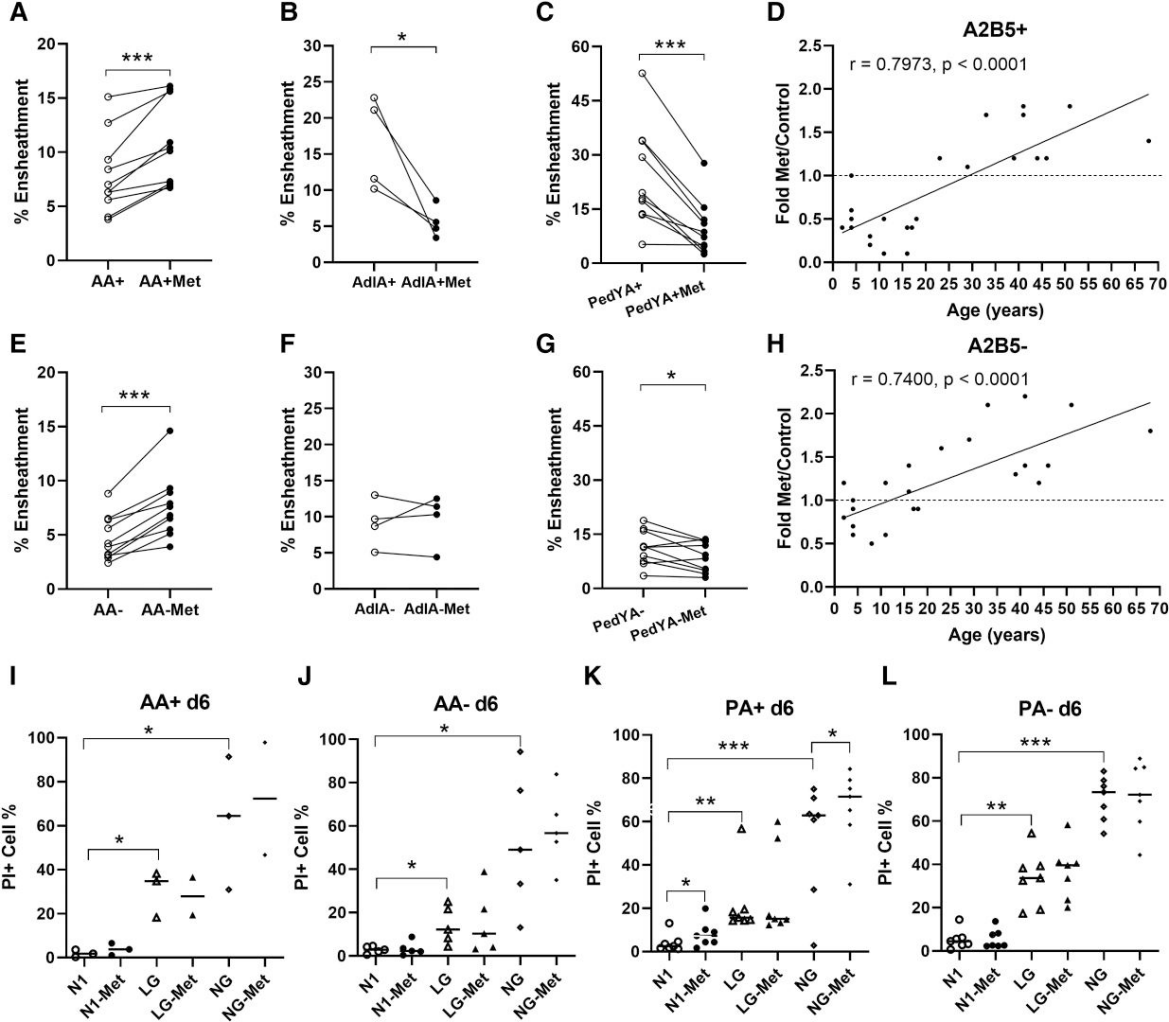
**Distinct cell type and age-related nanofibre ensheathment responses to metformin.** (**A–C**) Nanofibre ensheathment by A2B5+ (A+) cells from adult (**A**) (panel **A**), adolescent (adl) (panel **B**) and young paediatric (PedY) age samples (panel **C**). (**E–G**) Nanofibre ensheathment by mature OLs (A− cells) from adult (**A**) (panel **E**), adolescent (adl) (panel **F**) and young paediatric (PedY) (panel **G**) age samples. Data indicate the % of ensheathing cells by A+ and A− cells from individual donors under basal and metformin-added conditions. As in previous studies, under basal conditions, ensheathment by A+ cells was superior to that by A− cells for all age groups (adults—panel **A** 7.85 ± 1.16 versus panel **E** 4.7 ± 0.65, *n* = 10, **P < 0.01); paediatrics A+ 21.6 ± 3.3%; versus A− 7.8 ± 1.2%, *P* < 0.01 (combined Fig. 2B and C versus combined Fig. 2F and G), *n* = 14. Paediatric donor cells were superior to the matching adult-derived cells; A+ (panels **C**  **+**  **B** versus **A** **P < 0.01) and A− (panels **F**  **+**  **G** versus **E** ***P < 0.001). For adult-derived cells, the addition of metformin enhanced ensheathment for both A+ (panel **A**, 10.7 ± 1.2 versus 7.85 ± 1.2, ***P < 0.001) and A− cells (panel **E**, 7.6 ± 0.9 versus 4.7 ± 0.6, ***P < 0.001); for paediatric cells, there was a significant reduction in the presence of metformin versus control conditions for both sub-groups [PedY—9.7 ± 2.4 versus 23.7 ± 4.4%, *n* = 10, *P* < 0.001 (Fig. 2C); adolescents (adl) 5.6 ± 1.1 versus 16.4 ± 3.2%, *n* = 4, *P* < 0.05 (Fig. 2B)], with a similarly directed effect on A− cells (panel **G**, 11.3 ± 1.5 versus 8.7 ± 1.3, *P < 0.05). (**D** and **H**) Correlation between donor age and fold change in ensheathment for A+ cells (panel **D**) and A− cells (panel **H**) induced by metformin for the entire cohort (panel **D**: *r* = 0.7973, *P* < 0.0001; panel **H**: *r* = 0.74, *P* < 0.0001). (**I–L**) Propidium iodide staining was used to assess the cell viability of the dissociated cell culture. Data from dissociated cell cultures indicating the % propidium iodide-positive cells after 6 days in culture (d6) of (**M**) adult A+ cells (AA+); (**N**) adult A− cells (AA−); (**O**) combined paediatric/adolescent A+ cells (PA+); and combined paediatric/adolescent A− cells (PA−) under basal (N1) conditions or LG/NG conditions with or without addition of metformin (Met). *P < 0.05, **P < 0.01, ***P < 0.001.

We compared the extent of nanofibre ensheathment by A2B5+ cells (A+) and non-selected cells (A−) under basal conditions and in the presence of metformin across different age groups. Consistent with previous studies, under basal conditions, ensheathment by A+ cells was superior to that by mature cells (A−) for both age groups (Ped A+ 21.6 ± 3.3%; versus A− 7.8 ± 1.2%, *P* < 0.01 (combined Fig. 2B and C versus combined Fig. 2F and G); adult A+ 10.7 ± 1.2%; A− 4.7 ± 0.7%, *P* < 0.001 (Fig. 2A versus E). When the paediatric donors were considered in sub-groups of 12 years and under (PedY) versus adolescents (Adl) (age 13–18 years), we observed a trend for increased ensheathment by A+ cells from the younger group (Fig. 2C, 23.7 ± 4.4%, *n* = 10) compared to the adolescent group (Fig. 2B, 16.4 ± 3.2%, *n* = 4, *P* = 0.34). The overall extent of ensheathment for adult and paediatric A+ cells derived from these human brain-derived cells is less than that observed using postnatal rat-derived oligodendrocyte progenitor cells (Supplementary Fig. 1).

For adult-derived cells, the addition of metformin enhanced ensheathment for both A+ cells (10.7 ± 1.2 versus 7.8 ± 1.2, ***P < 0.001) (Fig. 2A) and A− cells (7.6 ± 0.9 versus 4.7 ± 0.6, ***P < 0.001 cells) (Fig. 2E). For A+ cells, there was a significant reduction in the presence of metformin versus control conditions for both paediatric sub-groups [PedY—9.7 ± 2.4 versus 23.7 ± 4.4%, *P* < 0.001 (Fig. 2C); adolescents (Adl) 5.6 ± 1.1 versus 16.4 ± 3.2%, *P* < 0.05) (Fig. 2B)]. A similar effect was observed on A− cells (8.7 ± 1.3 versus 11.3 ± 1.5, *P < 0.05) (combined Fig. 2F and G). There was an overall correlation between the fold change in ensheathment by A2B5+ and A− cells in response to metformin and the age of donors within the entire study cohort (Fig. 2D and H).

There was a marginally significant increase in the percentage of propidium iodide-positive cells in cultures of A+ cells from the paediatric cohort exposed to metformin compared to baseline values; no such effect was seen with any other cell type (Fig. 2I–L). All cell types showed a significant increase in the percentage of PI+ cells under LG and NG conditions, used as our ‘positive’ control.

### Molecular analysis of metformin-induced gene expression in adult and paediatric A2B5+ and A2B5− cells

Building upon our previous findings, we analysed the differential gene expression induced by metformin in cultured adult and paediatric A2B5+ (A+) and A2B5− (A−) cells, providing insights into their molecular responses to this therapeutic agent. We have previously reported the comparative molecular signatures of adult and paediatric mature OLs and A2B5+ cells based on bulk RNA sequencing of these cells within 24 h of isolation.^13^ For the current study, bulk whole-cell RNA sequencing was performed on cells after they were maintained in culture for 6 days to allow processes to grow out and then exposed or not exposed to metformin for 48 h.

**Figure 3:**
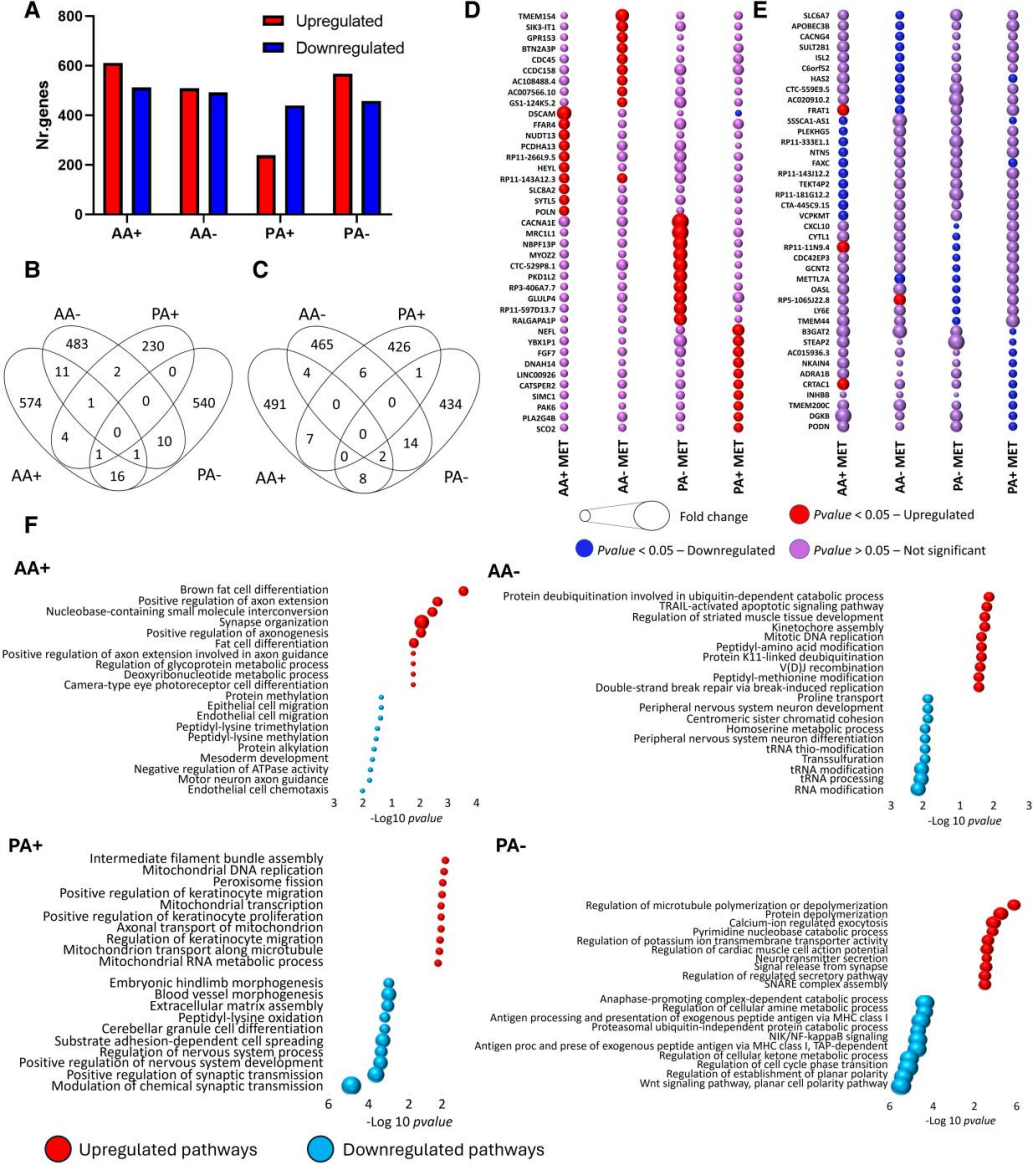
**Distinct cell type and age-related differences in gene expression induced by metformin.** (**A**) Bar graphs indicate there was a relatively greater proportion of down-regulated versus up-regulated genes for the paediatric A2B5+ cells (PA+), a pattern not observed for the adult A2B5+ cells or mature cells (AA−) from either age group. Up and down-regulation are based on paired *t*-test *P*-values <0.05. Red indicates the number of up-regulated genes, while blue indicates the number of down-regulated genes. (**B** and **C**) Venn diagrams indicating numbers of genes differentially increased (**B**) or decreased (**C**) respectively by metformin exposure. Up- and down-regulation are based on *P*-values <0.05. Fewer than 3% of genes for any cell type in response to metformin were shared with any other cell type. (**D** and **E**) Bubble plots showing the fold change in expression of the top 10 up- or down differentially regulated genes for each cell type with corresponding changes in the other cell types. Differential expression data are derived from *P*-values <0.05, normalized read counts >50 and fold change >1.2 for each cell population treated with metformin. (**F–I**) Ten most differentially up- and down-regulated pathways in response to metformin by the four cell populations based on the *P*-value <0.05. Up-regulated pathways for the AA+ cells (**F**) included those related to cell process outgrowth (e.g. axonal outgrowth, synapse formation) and lipid metabolism (fat cell differentiation).

Distinct differential gene expression induced by metformin for each cell type—There was a relatively greater proportion of down-regulated versus up-regulated genes for the paediatric A2B5+ cells (Fig. 3A), a pattern not observed for the adult A2B5+ cells or mature cells (A−) from either age group (Fig. 3A). Fewer than 3% of genes for any cell type in response to metformin were shared with any other cell type; this applied to both genes with differentially increased or decreased expression (Fig. 3A and B). Bubble plots showing the relative expression of the top 10 up- or down-differentially regulated genes induced by metformin for each cell type with corresponding expression in the other cell types (Fig. 3D and E).

Up-regulated pathways in adult A+ cells (Fig. 3F) encompassed cell process outgrowth, including axonal outgrowth and synapse formation, as well as lipid metabolism, specifically fat cell differentiation. Conversely, paediatric A+ cells exhibited down-regulated pathways associated with cellular morphology, development and synaptic transmission (Fig. 3F). Notably, paediatric A+ cells demonstrated up-regulated mitochondrial pathways, potentially indicating enhanced mitochondrial activity (Fig. 3F). However, no differentially regulated pathways related to cell death or cycling were identified across any cell type.

**Figure 4:**
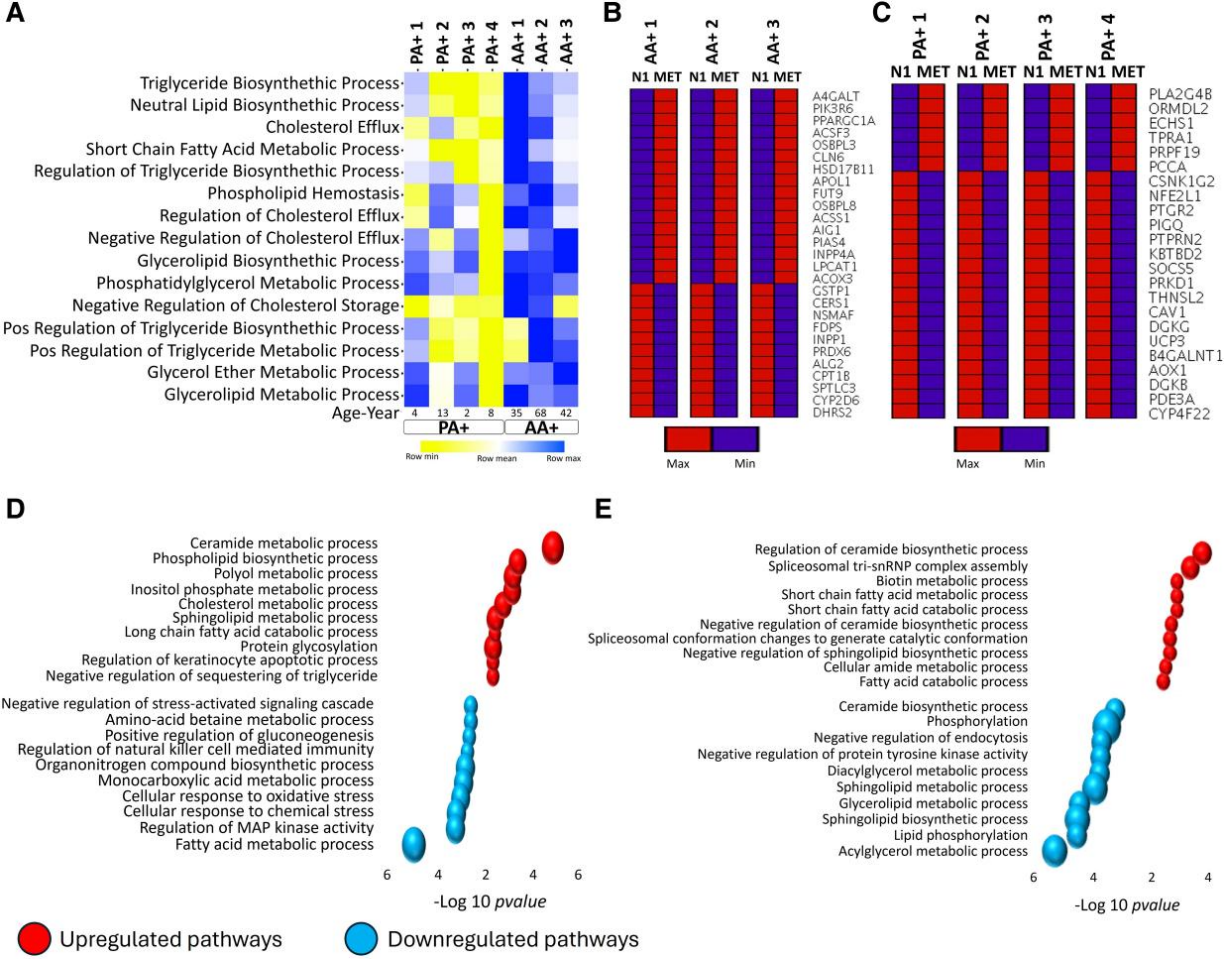
**Distinct cell type and age-related differences in lipid metabolism-related gene expression under basal conditions and in response to metformin.** (**A**) Enrichment analysis of AA+ and PA+ cells under basal condition. Enrichment scores for most pathways were greater for the adult versus paediatric A+ cells including for both triglyceride and neutral lipid biosynthesis pathways. (**B–C**) Heatmaps show the relatively greater proportion of lipid metabolism-related genes up-regulated in adult A+ cells versus paediatric A+ cells compared to control conditions upon metformin treatment. (**D**) Up- and down-regulated pathways for AA+ cells in response to metformin. (**E**) Up- and down-regulated pathways for PA+ cells in response to metformin.

Regarding lipid metabolism, a single sample gene set enrichment analysis revealed age-related differences in the properties of lipid metabolism between adult and paediatric A+ cells under basal culture conditions (Fig. 4A, Supplementary Fig. 2A and B). Enrichment scores for most pathways, such as triglyceride and cholesterol metabolism, were higher in adult A+ cells compared to paediatric A+ cells.

Regarding the response to metformin, there was no overlap in differentially expressed lipid metabolism-related genes among the various cell types. The heatmap demonstrated a relatively greater proportion of up-regulated genes in adult A+ cells compared to paediatric A+ cells (Fig. 4B, Supplementary Fig. 3A and B). Pathway analysis revealed up-regulation of lipid biosynthesis pathways in adult cells, whereas paediatric cells exhibited relative up-regulation of fatty acid catabolic pathways and negative regulators of lipid biosynthesis. Down-regulated pathways in the paediatric group included those involved in lipid biosynthesis (Fig. 4C and D).

**Figure 5:**
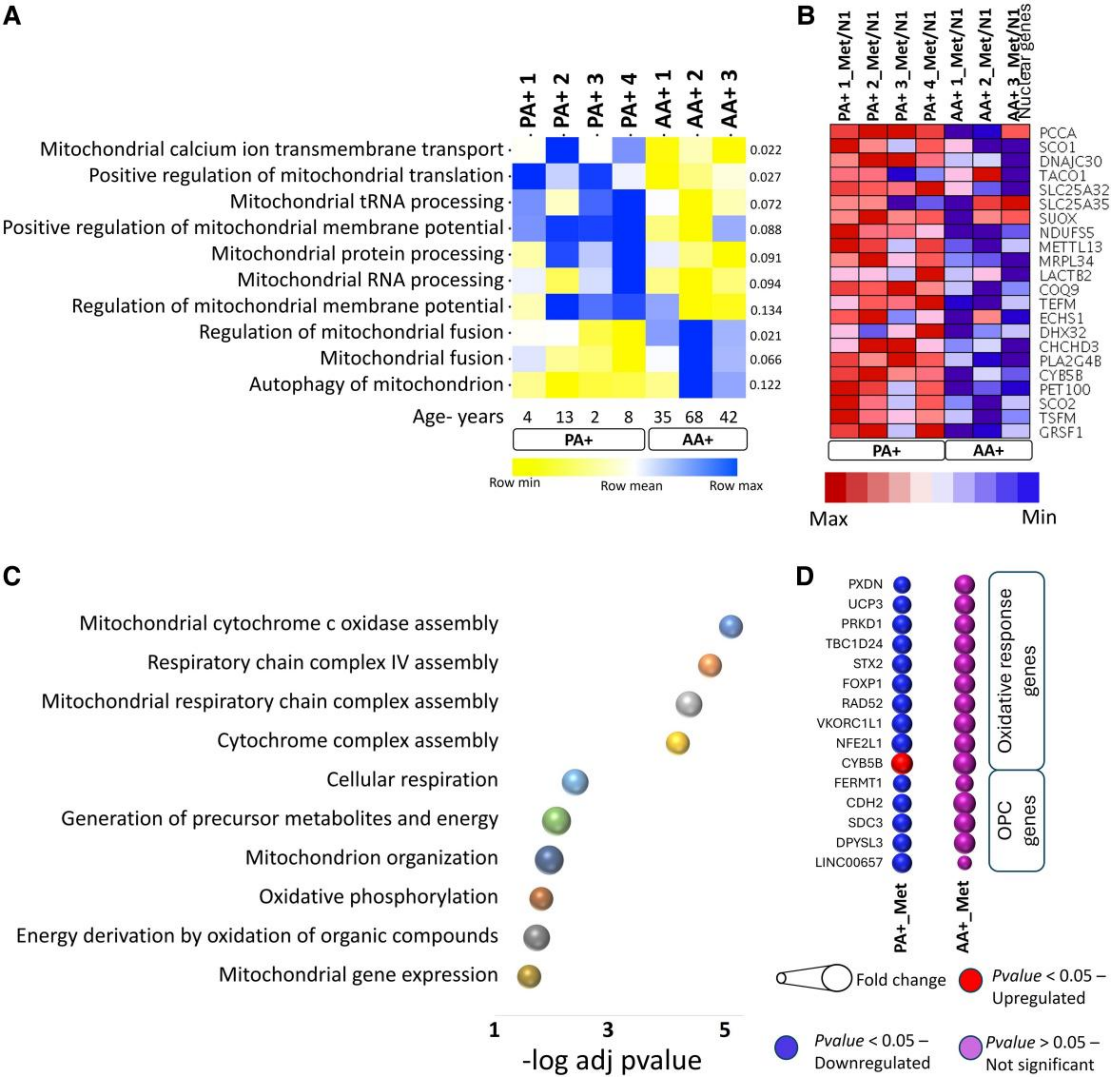
**Distinct cell type and age-related differences in mitochondria-related gene expression under basal conditions and in response to metformin.** (**A**) Enrichment analysis of AA+ and PA+ under basal conditions showed a higher enrichment score for mitochondrial transport and translation and a lower score for mitochondria fusion activity in PA+ cells. The heatmap (**B–C**) indicates the range of mitochondrial genes significantly changed by metformin in PA+ cells; all genes are up-regulated (*P* < 0.05). Pathways involved are shown in Panel **C**. Changes in gene expression in AA+ cells were not significant. (**D**) Bubble plot shows change in the expression of genes involved in protecting from oxidative stress and genes are expressed in oligodendrocyte progenitors. Expression of multiple genes were significantly down-regulated in PA+ cells. Paired t-tests were conducted on normalized read counts, with *P-*values <0.05 considered as statistically significant. Changes in gene expression in AA+ cells were not significant.

In terms of mitochondria gene expression, single sample gene set enrichment analysis under basal conditions revealed an enhanced enrichment score for mitochondrial transport and translation, along with a reduced score for mitochondria fusion activity in paediatric A+ cells (Fig. 5A). The heatmap indicated a range of significantly up-regulated mitochondrial genes in paediatric A+ cells treated with metformin (Fig. 5B). Pathways associated with mitochondria activity were up-regulated in paediatric A+ cells (Fig. 5C), while no significant changes were observed in adult A+ cells (Fig. 5C). Additionally, genes related to protecting against oxidative stress and cell differentiation were significantly down-regulated in paediatric A+ cells in response to metformin (Fig. 5D).

### Signalling pathway involvement and cellular response to metformin treatment: molecular and functional analyses

Given previous studies indicating that metformin effects on myelination are dependent on signalling via AMPK,^19^ we investigated the role of the AMPK pathway in the response of our various cell types to metformin. Molecular analysis revealed no changes in AMPK pathway gene expression in response to metformin across any of the cell types, nor were there differences in the mammalian target of rapamycin (mTOR) pathway.

**Figure 6:**
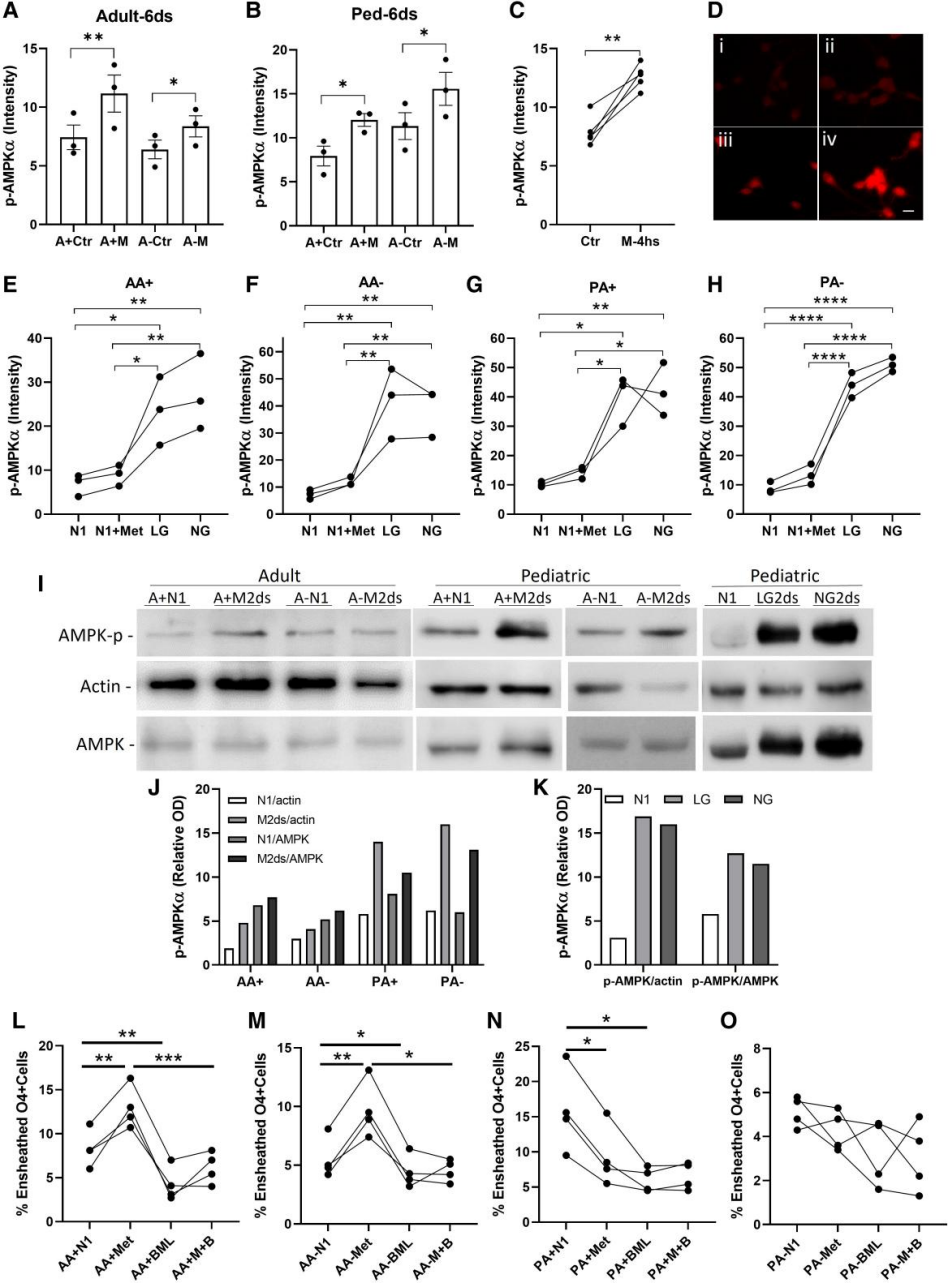
**Cell signalling responses of A2B5+ and A− cells to metformin.** Immunofluorescence—Panels **A** and **B**—for both adult and paediatric (Ped) samples, there was a significant increase in pAMPK immunofluorescence intensity at 6 days of exposure to metformin (**M**) for both A+ and A− cell types compared to control (Ctr) cultures. Panel **C**—increase in pAMPK immunofluorescence intensity at 4 h of exposure to metformin (data combines adult and paediatric samples, either A+ or A−). Panel **D** shows examples of immunofluorescence staining of pAMPK under N1(i), metformin (Met) treated (ii), LG (iii) and NG (iv) conditions. Scale bar = 20 µM. Panels **E**–**H**—for both paediatric and adult donors, increases in pAMPK immunofluorescence intensity at 48 h of exposure to metformin for both cell types were markedly less than observed under LG or NG conditions alone [AA+: N1 = 6.8 ± 1.4, N1 + Met = 8.9 ± 1.4 (1.3-fold of N1), LG = 23.6 ± 4.5 (3.5-fold of N1), NG = 27.2 ± 5.0 (4.0-fold of N1); AA−: N1 = 7.4 ± 1.0, N1 + Met = 11.9 ± 1.0 (1.6-fold of N1), LG = 41.8 ± 7.5 (5.7-fold of N1), NG = 38.93 ± 5.3 (3.3-fold of N1); PA+: N1 = 10.2 ± 0.5 (1.5-fold of AA + N1), N1 + Met = 14.4 ± 1.2 (1.4-fold of N1), LG = 39.9 ± 5.0), NG = 42.2 ± 5.2 (4.1-fold of N1); PA−: N1 = 8.9 ± 1.2 (1.2-fold of AA-N1), N1 + Met = 13.5 ± 2 (1.5-fold of N1), LG = 44 ± 2.5 (5.0-fold of N1), NG = 51 ± 2.5 (3.8-fold of N1)]. Panels **I–K** Western blots—increased ratio of pAMPK:total AMPK or actin at 2 days for both adult and paediatric derived A2B5+/− cells exposed to metformin compared to control conditions (cropped from the full length of blots as shown in Supplementary Fig. 5). Extent of up-regulation of pAMPK by metformin was markedly less than that observed for cells exposed to LG and NG conditions. The pAMPK levels in each condition were normalized to the corresponding bands of actin and total AMPK and are shown as relative activities of AMPK in Panels **J** and **K**. (**L–O**) addition of 1 µM BML-275 as an inhibitor of the AMPK pathway reduces the extent of nanofibre ensheathment by adult A+ (**L**) and A− (**M**) cells under control conditions and in the presence of metformin, as well as of paediatric A+ cells under control conditions (N1). There is no measurable effect on the reduced ensheathment induced by metformin on both paediatric A+ (**N**) and A− cells (**O**). *P < 0.05, **P < 0.01, ***P < 0.001—one-way ANOVA followed by Sidak’s multiple comparison tests except panel **C** with paired *t*-test.

Protein analyses involved assessing AMPK activity in response to metformin using immunofluorescence intensity and Western blot read-outs (Fig. 6). Addition of metformin produced a significant increase in the fluorescence intensity of pAMPK in adult and paediatric A+ and A− cells (Panels A and B at 6-day time point). Data on intermediate time points, Days 2 and 4, are now provided in Supplementary Fig. 4. As shown in Panel C, the metformin response could be observed within 4 h.

Panel D displays immunofluorescence staining examples under N1 (i), metformin-treated (ii), and LG (iii) and NG (iv) conditions.; the latter stress conditions were used as ‘positive’ controls, as we have previously reported.^29,30^ As shown in Panels E–H, the metformin-induced increase in pAMPK expression was considerably less pronounced compared to LG and NG conditions (range 1.2–1.6-fold of control versus 3.3–5.7-fold of control) in both adult and paediatric A+ and A− cells.

Western blot analysis revealed a detectable increase in the ratio of pAMPK to total AMPK after 2 days of metformin exposure in both paediatric and adult-derived A2B5+/− cells compared to control conditions (Panels I and J). However, the extent of pAMPK up-regulation induced by metformin was markedly lower than that observed in cells exposed to LG and NG conditions. (Panel K) (Supplementary Fig. 5).

Functional studies—The addition of BML-275/Dorsomorphin, an inhibitor of the AMPK pathway, resulted in a reduction in the extent of nanofibre ensheathment by adult A+ (Panel L) and A− (Panel M) cells under control conditions and in the presence of metformin. Similarly, paediatric A+ (Panel N) and A− (Panel O) cells under control conditions showed a decreased ensheathment when treated with BML-275. However, BML-275 had no measurable effect on the reduced ensheathment induced by metformin in paediatric cells.

### Comparison of the molecular signature of human and rodent A2B5+ cells

Through comparative analyses, we aimed to identify molecular signatures that distinguish between human and rat A2B5+ cells. We utilized our transcriptomic dataset and compared it with reported data on aged rat A2B5+ cells, which have been previously studied for their response to metformin.^19^ Our analysis revealed that human A2B5+ cells, whether examined upon isolation or after one week of maintenance in dissociated culture, displayed a more differentiated (mature) oligodendrocyte lineage molecular profile compared to their rat counterparts. Specifically, the human cells exhibited increased expression of mature oligodendrocyte genes and reduced expression of progenitor-linked genes such as CSPG4 and PDGFRα (Supplementary Fig. 1D).

## Discussion

In our study, we observed that metformin enhances ensheathment in adult-derived A2B5+ cells but reduces the response in the paediatric counterparts. Some positive effect is also noted on mature adult OLs with a reverse effect on the corresponding paediatric cells. Although we observe an overall significant correlation between ensheathment and age across our full cohort, our sample size is too small to identify the precise age of transition from negative to positive effect. Adult donors in our study were in the third to seventh decades, overlapping with the spectrum of secondary progressive multiple sclerosis but not reflecting an ‘aged’ population.

The hOL lineage cells used in this study were derived from ‘corridor’ tissue samples from surgical resections, providing cells suitable for both functional and molecular studies. O4+ A2B5+ cells were derived from the total OL lineage population by immunomagnetic bead separation with A2B5 antibody as used in previous human tissue-based studies^11,13^ and in the studies reported by Neumann *et al.*^19^ Previous immune-cytochemical and RNA sequencing studies indicate that the majority of human A2B5+ cells express mature OL cell markers.^11,12^ The human A2B5+ cells retain higher expression of progenitor markers (e.g. PDFGRα) than the non-selected cells we refer to as mature OLs. Our A2B5+ cells retain low levels of cycling as assessed by BrdU uptake.^31^ The A2B5+ cells used in our study differ from A2B5+ cells in the fetal brain; the latter are multi-potent cells with only a rare minority expressing even early OL progenitor markers before the late second trimester.^32^ Neumann *et al.*^19^ found that A2B5+ cells of both young and aged rats co-expressed PDGFRα but did not express mature lineage markers (CNPase and MBP). Using our data from Luo *et al.*^13^ and data from Neumann *et al.*,^19^ we provide a direct comparison indicating the human A2B5+ cells are further along the OL lineage compared to adult rat brain-derived A2B5+ cells.

As our molecular analyses showed distinct profiles of each of our cell populations under ‘control’ conditions, we examined the effects of metformin using paired analyses for each cell type for each age group. We observed that each cell type showed a distinct profile of genes differentially expressed in response to metformin with very limited overlap between groups. For the adult A2B5+ cells, we observed up-regulation of pathways relevant for process outgrowth and cell metabolism consistent with our functional studies of metformin showing enhanced ensheathment. For the paediatric A+ cells, the most apparent was the overall relatively greater proportion of genes with reduced versus increased expression compared to their adult counterparts. Down-regulated pathways for the paediatric A+ cells included pathways involved in cell morphology, development and synaptic transmission, consistent with the observed reduced ensheathment by metformin.

We focused on lipid expression gene expression as the myelin membrane of OL is highly enriched in lipids with substantial rates of turnover. Our comparative analysis of gene expression related to lipid metabolism indicated that under basal conditions the adult cells showed enhanced expression of genes involved in lipid biosynthesis with further up-regulation of such pathways upon metformin exposure. For paediatric cells, there was relative up-regulation of fatty acid catabolic pathways and negative regulators of lipid biosynthesis.

Our analysis also indicated differential expression of mitochondrial genes between paediatric and adult cells under basal and metformin conditions. Paediatric A2B5+ cells under basal conditions show enhanced enrichment scores for mitochondrial transport and translation and reduced scores for mitochondria fusion activity. Only the paediatric A2B5+ cells showed significant up-regulation of mitochondrial genes in response to metformin. Metformin is variably reported as enhancing or inhibiting mitochondrial respiration, the latter via either complex 1 or 4, dependent on the cell type.^33-36^ Neumann *et al.* documented that metformin-enhanced mitochondrial respiration in their rat oligodendrocyte progenitor cells, contributed to cell differentiation. Augmented mitochondrial gene expression may also indicate an element of cell stress^37^ in line with the marginal increase in per cent of cell death we noted in the metformin-exposed paediatric A2B5+ cells. The paediatric A+ cells showed down-regulation of genes related to protecting from oxidative stress.

Metformin effects are most attributed to its actions on AMPK although independent pathways are also described.^36,38,39^ Using both immunofluorescence and western blot data, we show that metformin-induced measurable AMPK activation in all our cell types but significantly less than that induced by LG and NG conditions that were used as ‘positive controls’. The LG and NG conditions result in a rapid reduction of ATP levels and AMPK activation.^30^ Neumann *et al*.^19,40^ attributed the effect of starvation and metformin on promoting myelination in aged animals to signalling via the AMPK pathway. Consistent with the latter, we observed that inhibiting AMPK reduced the ensheathment-promoting effect of metformin on our adult cells. AMPK induction can also inhibit the downstream mTOR pathway with a resultant reduction in protein synthesis, as we have shown in our previous studies in which we exposed the human mature OLs and A+ cells to LG and NG conditions.^28,29^ As mentioned, we observe a disproportionate ratio of down versus up-regulated genes in our paediatric A2B5+ cells.

## Conclusion

Our results emphasize the need to consider species, age, and differentiation stage of OL lineage cells when evaluating the effect of metformin on the myelination process. The study of Neumann *et al*.^19^ also indicates the need for consideration of sex-based responses. Clinical trials aimed at myelin restoration across the age spectrum will indicate how these differences translate *in vivo*.

## Supplementary Material

fcae109_Supplementary_Data

## Data Availability

All bulk RNA-seq samples utilized in this study have been deposited in the Gene Expression Omnibus of NCBI with GSE247159 number.
