# Supplementary material for: Age-dependent effects of metformin on human oligodendrocyte lineage cell ensheathment capacity
Source: Brain Commun. 2024 Mar 28;6(2):fcae109. doi: 10.1093/braincomms/fcae109 (PMC11005772; doi:10.1093/braincomms/fcae109)
Supplement: fcae109_Supplementary_Data [file fcae109_supplementary_data.zip › Supplementary figures.docx]

**Supplementary figures**

**Supplementary Figure 1.**
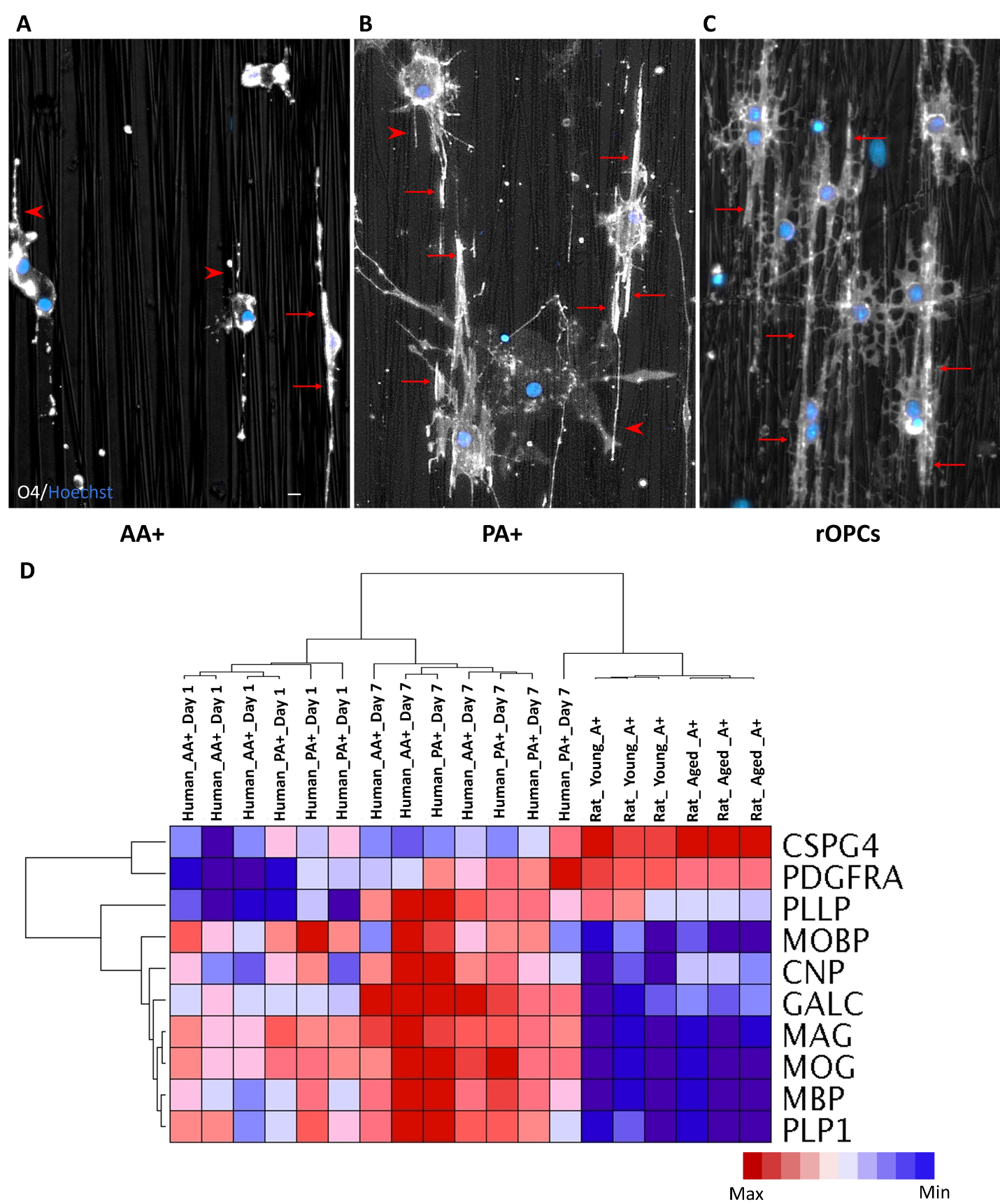


**Supplementary Figure 1.** **Nanofiber Ensheathment by A2B5+ Cells from human Adult and Pediatric Donors, and Rat Postnatal oligodendrocyte progenitor cells (OPCs).** (A-C) Immunofluorescence staining examples demonstrating the ensheathment of nanofibers by A2B5+ cells derived from human adult (AA+) (A) and pediatric (PA+) (B) donors, as well as rat postnatal OPCs (C). Cells were immunostained with O4 (Alexa Fluor 647) for identifying ensheathed segments and Hoechst 33342 (blue) for nuclear staining. Arrows indicate ensheathed segments, while arrowheads indicate processes that do not ensheath the nanofibers. Scale bar = 10 μm.

(D) Comparative analyses of the molecular signature of human adult and pediatric A2B5+ cells with reported data of aged adult rodent A2B5+ cells. The human A2B5 cells express a more differentiated (mature) OL lineage molecular profile than do their rat counterparts i.e. increased expression of mature OL genes and reduced expression of progenitor-linked genes (CSPG4, PDGFRα). Hierarchical clustering was conducted on the normalized read counts and Pearson correlation was used to calculate both genes and sample distances.

**Supplementary Figure 2.
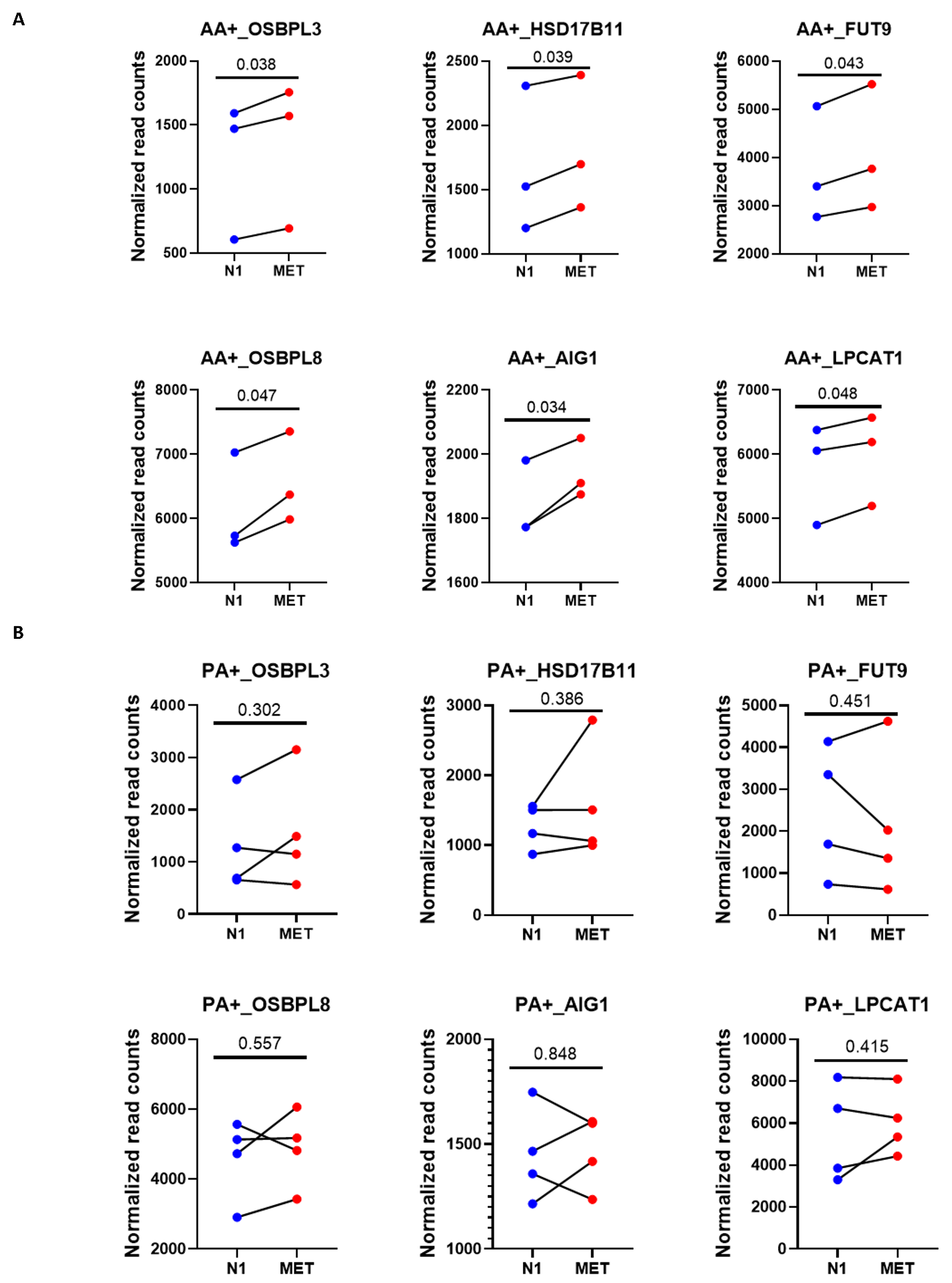
**

**Supplementary Figure 2.** **Gene Expression Changes in Lipid Metabolism Pathway in Adult A+ and Pediatric A+ Cells upon Metformin Treatment.** (A) Gene expression plots showing upregulated genes involved in lipid metabolism in adult A+ cells upon metformin treatment. (B) Gene expression pattern of the same six genes involved in lipid metabolism in pediatric A+ cells. None of these genes exhibited significant upregulation in pediatric A+ cells. Paired t-tests were conducted on normalized read counts, with *p-values* less than 0.05 considered as statistically significant. Each dot on the plots represents a sample from an individual donor.

**Supplementary Figure 3.**


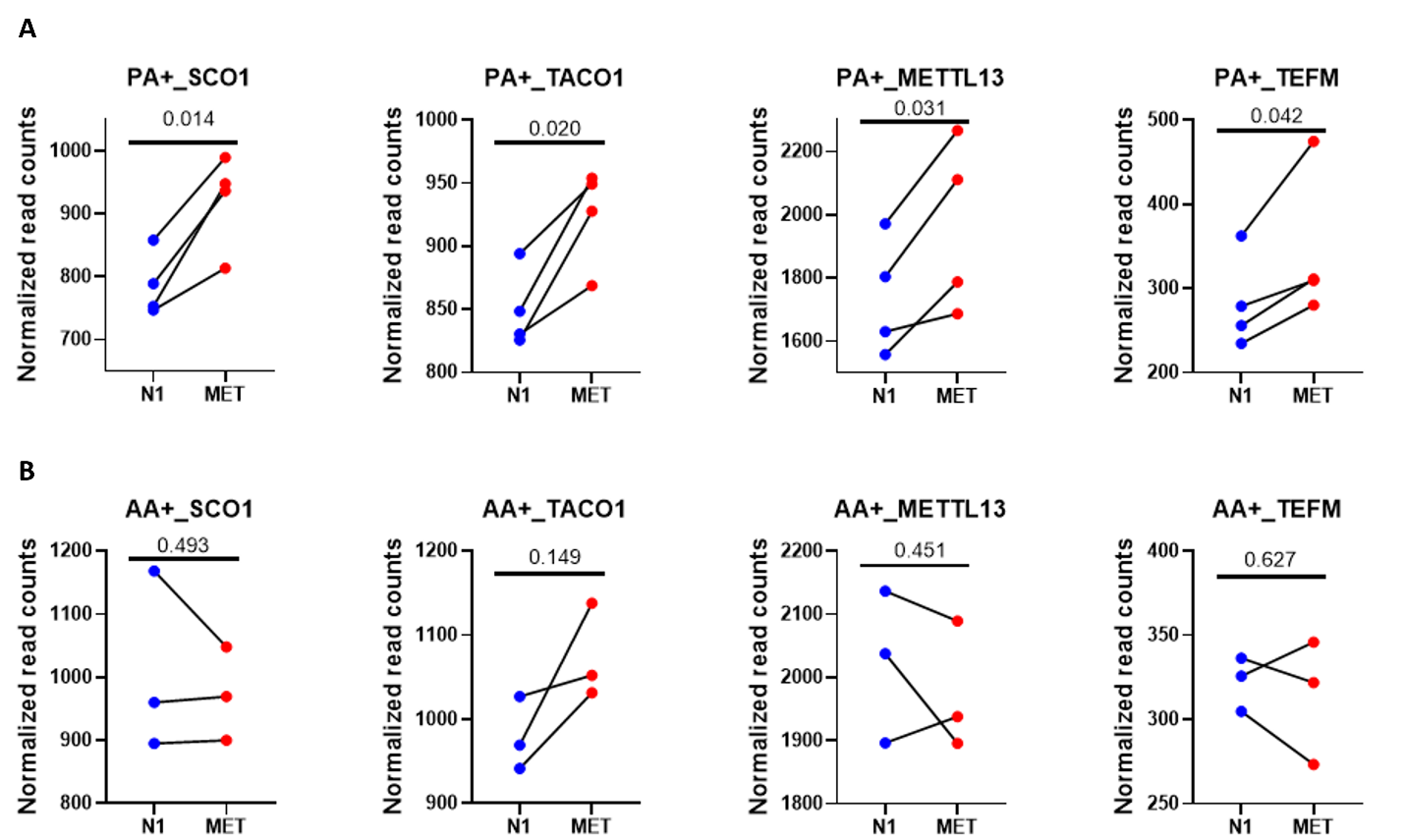


**Supplementary Figure 3.** **Gene Expression Changes in mitochondrial genes in Adult A+ and Pediatric A+ Cells upon Metformin Treatment.** (A) Gene expression plots showing upregulated genes involved in mitochondrial processes in pediatric A+ cells upon metformin treatment. (B) Gene expression pattern of the same four genes involved in mitochondrial processes in adult A+ cells. None of these genes exhibited significant upregulation in adult A+ cells. Paired t-tests were conducted on normalized read counts, with *p-values* less than 0.05 considered as statistically significant. Each dot on the plots represents a sample from an individual donor.

**Supplementary Figure 4.**


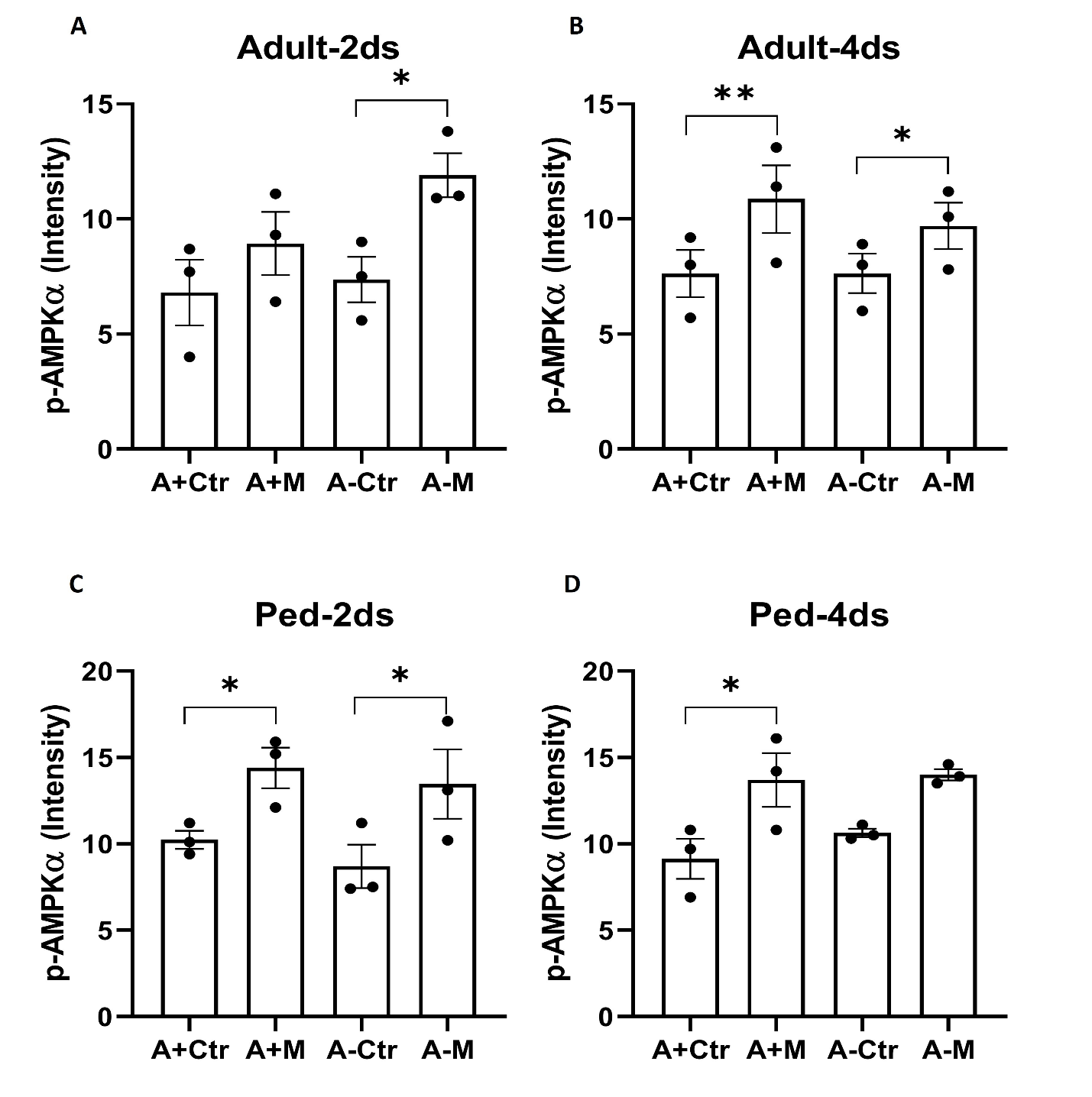


**Supplementary Figure 4.** **For adult A+ and A- cells at days 2 and 4 (Panels A and B) and pediatric (Ped) samples (Panels C and D), there was a significant increase in pAMPK immunofluorescence intensity at time points shown with exposure to metformin (M) compared to control (Ctr) cultures.**

*p<0.05, **p<0.01, one-way ANOVA followed by Sidak’s multiple comparisons test.

**Supplementary Figure 5.**


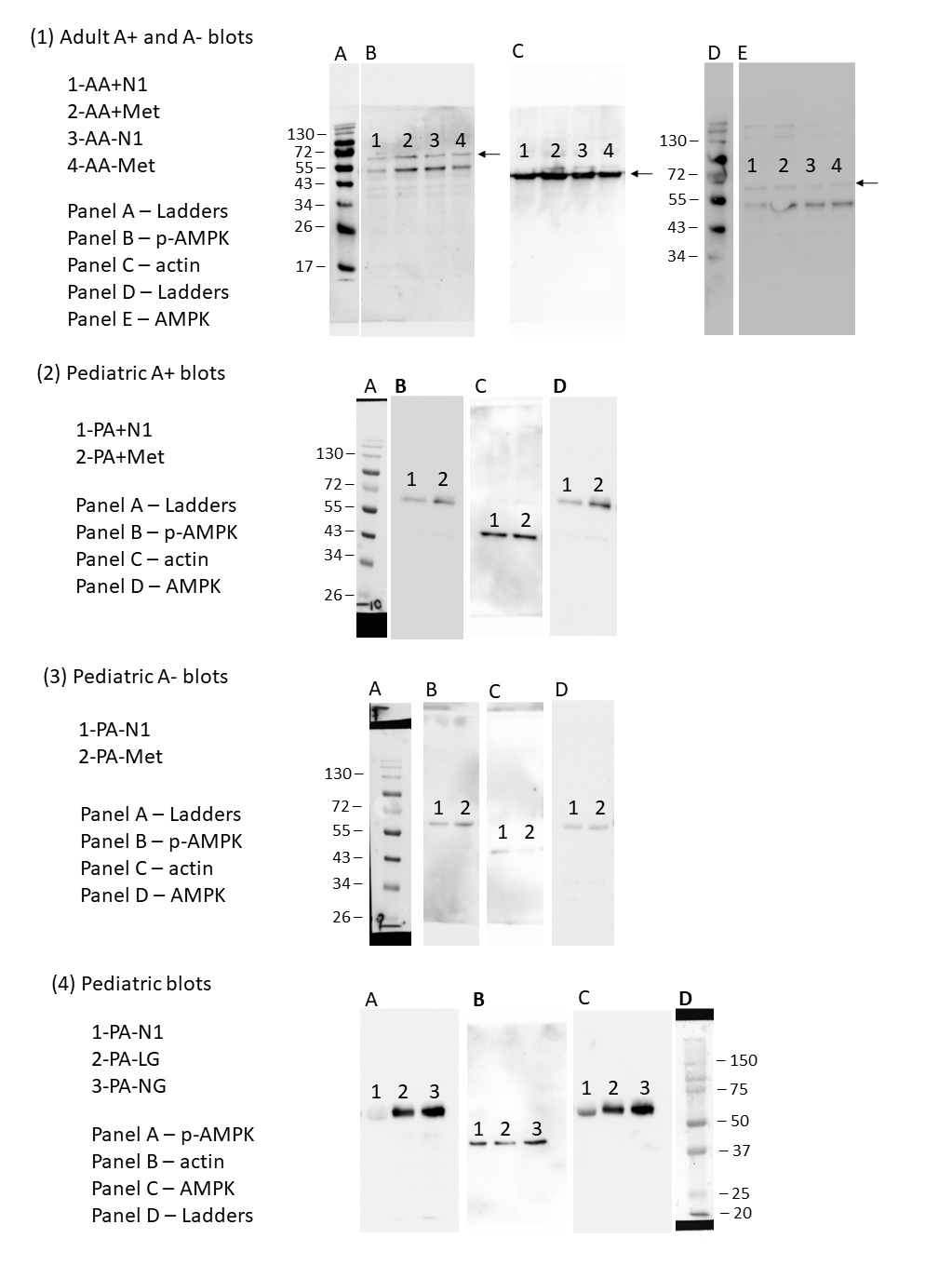


**Supplementary Figure 5. Full length of Western blots of Phosphorylated AMPK changes in Adult A+/- and Pediatric A+/- cells upon Metformin treatment for 2 days for Figure 6I**

(1) Adult A+ and A- cells were treated with or without metformin. Phosphorylated AMPK was detected by p-AMPK antibody (Phospho-AMPK alpha-1,2 (Thr183, Thr172) Recombinant Rabbit Monoclonal Antibody (10H2L20), Invitrogen, # 701068) (Panel B). Total actin was detected by actin antibody (monoclonal Anti-actin antibody, A4700, Sigma) (Panel C). Total AMPK was detected by AMPK antibody (AMPKα(D5A2) Rabbit mAb, #5831, Cell signaling Technology) (Panel E). Panel E involves the same samples as used in panels A and B, but in a different gel due to usage of multiple antibodies). Arrows in panels B, C and E indicate the corresponding proteins. Molecular size markers are shown in panel A and D.

(2) Pediatric A+ cells were treated with or without metformin. Phosphorylated AMPK was detected by p-AMPK antibody (Phospho-AMPK alpha (Thr172) (40H9) rabbit mAb, #2535, Cell signaling Technology) (Panel B). Total actin was detected by actin antibody (monoclonal Anti-actin antibody, A4700, Sigma) (Panel C). Total AMPK was detected by AMPK antibody (AMPKα(D5A2) Rabbit mAb, #5831, Cell signaling Technology) (Panel D). Molecular size markers are shown in panel A.

(3) Pediatric A- cells were treated with or without metformin. Phosphorylated AMPK was detected by p-AMPK antibody (same as in #(2)) (Panel B). Total actin was detected by actin antibody (same as in #(2)) (Panel C). Total AMPK was detected by AMPK antibody (same as in #(2)) (Panel D). Molecular size markers are shown in panel A.

(4) Pediatric A- cells were cultured under N1, LG and NG conditions for 2 days. Phosphorylated AMPK was detected by p-AMPK antibody (same as in #(2)) (Panel A). Total actin was detected by actin antibody (same as in #(2)) (Panel B). Total AMPK was detected by AMPK antibody (same as in #(2)) (Panel C). Molecular size markers are shown in panel D.
