# Supplementary material for: Age-dependent effects of metformin on human oligodendrocyte lineage cell ensheathment capacity
Source: Brain Commun. 2024 Mar 28;6(2):fcae109. doi: 10.1093/braincomms/fcae109 (PMC11005772; doi:10.1093/braincomms/fcae109)
Supplement: fcae109_Supplementary_Data [file fcae109_supplementary_data.zip › Supplementary Table 1.docx]

**Supplementary Table 1. Clinical details of samples used RNAseq and functional assays.**

| **Patient#** | **Age** | **Sex** | **Nanofiber** | **PI** | **Protein** | **RNA** |
| --- | --- | --- | --- | --- | --- | --- |
| **Pediatric** |  |  |  |  |  |  |
| 1 | 1.5 years | M | + |  |  |  |
| 2 | 2 years | M | + | + |  | + |
| 3 | 4 years | F | + |  |  |  |
| 4 | 4 years | M |  | + |  |  |
| 5 | 4 years | M | + |  |  | + |
| 6 | 4 years | M | + |  |  |  |
| 7 | 4 years | M |  |  | + |  |
| 8 | 4years | F | + |  |  |  |
| 9 | 8 years | M | + |  |  |  |
| 10 | 8 years | F | + |  | + | + |
| 11 | 11 years | M | + | + | + |  |
| 12 | 11 years | M | + | + | + |  |
| 13 | 12 years | F |  |  | + |  |
| 14 | 13 years | F |  |  |  | + |
| 15 | 15 years | M |  | + |  |  |
| 16 | 15 years | F |  |  | + |  |
| 17 | 16 years | F | + | + | + |  |
| 18 | 16 years | M | + | + | + |  |
| 19 | 17 years | F | + |  |  |  |
| 20 | 17 years | M |  | + |  |  |
| 21 | 18 years | M | + |  | + |  |
|  |  |  |  |  |  |  |
| **Adult** |  |  |  |  |  |  |
| 22 | 23 years | F | + | + | + | + |
| 23 | 29 years | F | + | + | + |  |
| 24 | 33 years | M | + |  |  |  |
| 25 | 35 years | M |  | + | + |  |
| 26 | 35 years | M |  |  |  | + |
| 27 | 37 years | F |  |  | + |  |
| 28 | 39 years | M | + |  |  |  |
| 29 | 41 years | F | + |  |  |  |
| 30 | 44 years | M | + |  |  |  |
| 31 | 44 years | M | + |  |  |  |
| 32 | 44 years | F |  |  | + |  |
| 33 | 46 years | M | + |  |  | + |
| 34 | 51 years | F | + |  |  |  |
| 35 | 57 years | M |  | + |  |  |
| 36 | 68 years | M | + |  |  | + |
| 37 | 73 years | F |  | + |  |  |
